# The influence of physical activity on mental well-being in college students: a systematic review

**DOI:** 10.3389/fpsyg.2025.1573446

**Published:** 2025-05-20

**Authors:** Ruochen Li, Rong Huang

**Affiliations:** Faculty of Sports, Shaanxi University of Technology, Hanzhong, China

**Keywords:** college students, well-being, physical activity, good mood, subjective well-being

## Abstract

**Introduction:**

Physical activity (PA) is widely recognized for its benefits on mental well-being, yet evidence regarding its specific impact on college students remains insufficient. This systematic review investigates the association between PA and mental well-being in college students, focusing on key outcomes such as self-esteem, depression, and subjective well-being.

**Methods:**

A comprehensive search was conducted in Web of Science and Scopus up to August 2024 using Boolean operators (e.g., “physical activity,” “mental well-being,” “college students”). Inclusion criteria targeted studies with quantitative or qualitative data on PA and well-being outcomes in higher education populations. After screening 746 abstracts and applying exclusion criteria (e.g., non-college populations, tangential PA focus), 28 articles were selected for full-text review following PRISMA guidelines.

**Results:**

The review synthesized findings across three dimensions: (1) PA positively correlates with self-esteem, though gender and cultural biases may influence outcomes; (2) weak negative correlations (*r* < 0.2) were observed between PA and depression, indicating PA’s potential but non-exclusive role; (3) PA enhances subjective well-being, particularly through intrinsic motivation and structured activities. However, heterogeneity in well-being definitions and measurement tools limited comparability.

**Discussion:**

While PA demonstrates a positive association with mental well-being in college students, inconsistencies in study designs and mechanistic explanations highlight the need for future research to clarify causal pathways, differentiate PA types/intensity, and standardize well-being metrics. Universities should prioritize PA promotion to support student well-being during this critical developmental stage.

## 1 Introduction

The relationship between physical activity and mental well-being has increasingly come under the spotlight over recent years. With much literature analyzing the role of physical activity on mental well-being, a growing number of valuable research results have been obtained, such as the influence of PA on self-development (Molina-García et al., 2011), the improvement of sleep quality (Hadas and Joseph, 2022), and the beneficial effects of PA on good mood (Hou, 2022). While these findings highlight the broad psychological benefits of PA across populations, emerging scholarship underscores the need to contextualize these effects within specific life stages.

College is a crucial period in which young adults adjust to a new environment (Bodziony and Stetson, 2024; Molina-García et al., 2011). However, understanding the determinants of physical activity in this population is important for developing targeted interventions. Factors such as motivation, time management, social support, and access to facilities may significantly influence PA engagement among college students. While the positive influence of physical activity on mental well-being has become a consensus, the impact of PA on well-being in the context of college student groups have yet to be comprehensively addressed (Peralta et al., 2021; Yuliastrid et al., 2024). As the prevalence of mental and physical health difficulties among college students grow (Lee et al., 2023; Zhou and Guo, 2023), it is critical to understand the positive influence of physical activity.

A few studies have examined the association between physical activity and well-being. Peralta et al. (2021) adopted questionnaires to collect attitudes toward well-being in six dimensions from 3,143 European university students (1,456 men and 1,687 women) from 27 countries. The evidence shows that students who practice PA more often are associated with better well-being.

Although the study by Miguel Peralta confirmed the existence of a positive association between PA and well-being, it did not provide plausible causal explanations for the relationship between the two variables. Based on cross-sectional nature, the available data were not sufficient to draw firm conclusions about physical activity improving students’ mental well-being. Therefore, we should collect more detailed information about the type, intensity, and duration of physical activity. For example, not only record whether a student engages in physical activity but also distinguish between different types such as aerobic exercise, strength training, or flexibility exercises. This richer data set would help to better understand the complex relationship between physical activity and well-being and provide more clues for causal inference.

In addition, using questionnaires to assess Participation in PA and Health perception could be subject to bias. On the one hand, data were self-reported rather than objectively measured. The variation of individual referents may influence the accuracy of self-reporting both frequency and intensity of physical activity (Pašková et al., 2019). On the other hand, the study did not provide objective evaluation criteria about whether students’ mental well-being was truly improved. Both of these issues prevented us from exploring the direct evidence of how PA and well-being interact.

Furthermore, the problem of the unexplained use of the term “well-being” appears several times in Peralta et al. (2021)’s study. Such as “well-being and health perceptions” Peralta et al. (2021, p. 45) “physical and psychological well-being” Peralta et al. (2021, p. 42). The article by Peralta et al. (2021) does not clarify the definition of well-being and its derivatives. This omission may lead future researchers to foster unclear thinking by using “mental well-being” in inaccurate or vague ways. The author should explicitly define what “well-being” means in the context of their study. In addition, when using derivatives such as “mental well-being,” “physical and psychological well-being,” they should also provide specific explanations of the components and dimensions included in these concepts. By doing so, readers can better understand the different aspects of well-being that the authors are referring to and how they are related to each other.

Unfortunately, the research community has yet to reach a consensus on how well-being should be defined. Due to a lack of agreement on the concept of well-being, measurement strategies of well-being varied across all studies. In Blažević et al. (2021) study, well-being is measured by three factors: positive well-being, depression/anxiety, and good mood. Hou (2022) suggested that well-being is reflected in three dimensions: positive emotion, negative emotion, and life satisfaction. With the different definitions and measurements, we cannot efficiently explain the effectiveness of various interventions. Taking these concerns into account, the purpose of this study was to identify the different ways to define the concept of mental well-being and investigate the mental function of physical activity. To that end, the study aimed to provide an answer to the following questions:

What is the mental well-being?

What’s the impact of physical activity on mental well-being?

## 2 Methods

### 2.1 Search strategy

This research uses the Web of Science and Scopus databases to search articles closely related to mental well-being and physical activity, including studies published up to August 2024. Using Boolean operators (“OR” and “AND”) to refine our search. To capture a comprehensive sample of relevant articles, we used multiple search terms relating to “physical activity”, “mental wellbeing” and “college”. These keywords were derived from the literature and refined following a trial search in each database. For “physical activity,” we have borrowed from a series of keyword variations (e.g., physical fitness, physical inactivity) (Hallal et al., 2006). For “mental well-being”, we use keyword variations (e.g., well-being, well-being) (Rose et al., 2017).

The final search query was structured as follows:

We only included study populations with higher education background, for example, studies on old age populations were excluded.

### 2.2 Inclusion criteria

We focus on studies that assess the association between physical activity and mental well-being among college students; studies were eligible if they provided quantitative data regarding university students’ well-being outcomes as a function of physical activity or used qualitative methods to collect and analyze data. This criterion ensures that the content of the studies is directly relevant to our research topic. It helps in filtering out studies that mention physical activity and well-being tangentially but do not contribute significantly to its understanding. We have also included studies that reported on a component of mental well-being and physical activity to avoid potentially relevant studies being overlooked. Mental well-being, as a multidimensional concept, has differing meanings (Ryff, 1989). We included studies that used the concept of well-being related to how individuals experience their quality of life.

After removing duplicates (*n* = 374), studies not in the English language (*n* = 56), book chapters (*n* = 10), and conference papers (*n* = 29), 746 abstracts were screened based on inclusion and exclusion criteria. We excluded studies on a thematic basis if physical activity was not treated as an independent variable, if physical activity was not associated with a well-being outcome or if the target population was not college students (*n* = 714).

**Figure 1:**
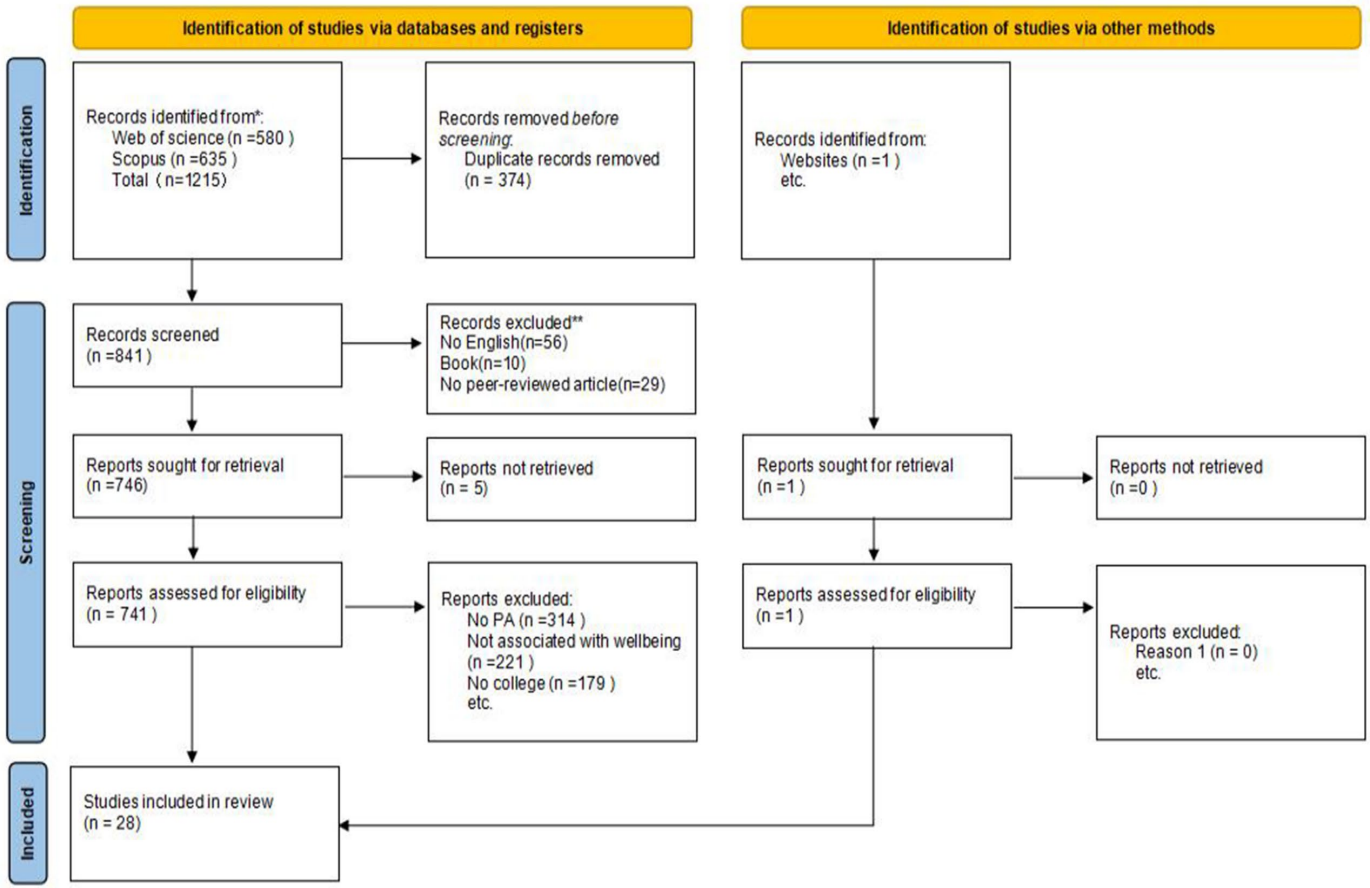
PRISMA 2020 flow diagram for new systematic reviews which included searches of databases, registers, and other sources.

Guided by inclusion and exclusion criteria, we collected a representative collection of 28 currently available studies Full-text versions of the articles were obtained, with each article being reviewed and confirmed as appropriate by the authors. The process of article selection followed the Preferred Reporting of Items for Systematic Reviews and Meta-Analyses (PRISMA) Statement. The flow diagram (Figure 1) illustrates the literature selection process.

### 2.3 Data extraction analysis

The extracted data from 28 studies included in this systematic review are summarized in Table 1 (see Supplementary materials). The table systematically presents key information of the selected literature, including: author, geographical location, journal title, study design, research participants, data collection, data analysis techniques, and findings.

**Table 1:** Data extraction.

Step	Terms	Results
Database		
Web of science		
1	TS = (“mental well-being”OR“well-being”OR“wellbeing”)	262,165
2	TS = (“physical activity”OR“physical exercise”OR “physical fitness”OR“physical inactivity”)	304,215
3	TS = (“university students” OR “college students” OR “undergraduate students”)	167,218
4	TS = (“higher education” OR “universit*” OR “college*”)	1,358,441
5	1 AND 2 AND 3 AND 4	580
Scopus		
1	TITLE-ABS-KEY (“mental well-being” OR “well-being” OR “wellbeing” OR “well-being”)	385,098
2	TITLE-ABS-KEY (“physical activity” OR “physical exercise” OR “physical fitness” OR “physical inactivity”)	382,808
3	TITLE-ABS-KEY (“university students” OR “college students” OR “undergraduate students”)	228,502
4	TITLE-ABS-KEY (“higher education” OR “university*” OR “college*”)	2,535,652
5	1 AND 2 AND 3 AND 4	635

## 3 Results

### 3.1 Q1 what is the mental well-being?

In this chapter, we begin by reviewing the two principal approaches to defining well-being: the hedonic and eudaimonic approaches. We then discuss subjective well-being, psychological well-being, and other conceptions of well-being.

Over the past few decades, there has been considerable debate about the definition of well-being. Due to its multifaceted nature, concepts of well-being have been proposed that are not consistently included in either subjective or objective conceptions of well-being (Blažević et al., 2021; Chen et al., 2013; Deci and Ryan, 2008). Evidence thus far seems to suggest that it cannot be fully represented by any one construct.

Although subjective well-being has commonly been interpreted as mental well-being, this approach also has some limitations. Based on reviews of the literature on students’ well-being, we want to reconstruct the definition of well-being from the perspective of integration. Regardless of whether these papers focused on multiple or single definitions, each paper included in the review reflected the following claims about the definition of mental well-being.

Mental well-being is an individual’s positive evaluation of their life, integrating both subjective experiences and objective conditions. It encompasses cognitive assessments of life satisfaction, emotional states including positive and negative emotions, and psychological functioning of personal development.

This comprehensive definition acknowledges that well-being is influenced by a combination of personal perceptions and objective circumstances, reflecting both hedonic and eudaimonic aspects of well-being.

We illustrate each of these claims here by referencing a range of papers from different contexts and focusing on different interpretations. For example, Blažević et al. (2021) noted that subjective theory implies an individual’s self-reflection on their life, including respecting their own preferences for certain aspects of well-being. The term “own preferences” indicates well-being is considered subjective because this internalized self-reflection does not grant to the external judgments of psychologists (Blažević et al., 2021, p. 111). Blažević’s view also responds to the fact that well-being can be described as a cognitive dimension that is associated with one’s evaluations of their lives.

Chen et al. (2016) further explain the definition from the perspective of cognition. For instance, he noted that subjective well-being refers to how people think and feel about their life experiences in positive ways. Chen’s point indicates that well-being is a positive psychological experience. Over the past few years, psychology’s focus on the amelioration of psychopathology overshadowed the positive aspect of well-being. Well-being was defined as an operational definition limited to the absence of negative psychological symptoms (Yazici et al., 2016). We argue that the term “well-being” should focus on positive experiences rather than negative experiences, namely, well-being rather than ill-being.

According to Chen et al. (2016), life satisfaction as a cognitive component of subjective well-being has been widely accepted. However, life satisfaction is not strictly a subjective concept. Instead, objective measures need to be considered to determine materialistic living. For example, Pašková et al. (2019) noted that life satisfaction is connected with objective conditions of life of a person, such as income, type of living, and rate of social contacts. The evidence shows that both objective conditions and subjective conditions are strongly related to the general construct of cognitive evaluation.

Although the emphasis on cognitive aspect by subjectivist is commendable, psychologists who have adopted the eudaimonic view consider well-being to consist of more than just cognitive evaluation (Martela and Sheldon, 2019). For instance, Bartley and Belgrave (1987) defined psychological well-being by some indicators that reflect the psychological function of well-being, such as self-esteem and assertive behavior. From this perspective, well-being is not only about whether people are feeling well but also involves certain qualities that are essential for people seek to improve their well-being.

In Bartly and Belgrave’s view, well-being is conceptualized in terms of some set or combination of psychological elements. However, there is no consensus on which elements best represent psychological well-being, and the conceptual structure of psychological well-being is still unstable.

### 3.2 Q2 What’s the impact of physical activity on mental well-being?

The second aim of this review was to determine the relationship between physical activity and well-being. Due to a lack of agreement across the concept and measurement of well-being, we include conclusions for various aspects of the target population that contribute to our understanding of the relationship between PA and well-being. For example, well-being and its derivatives, such as subjective well-being, positive well-being, or psychological well-being, are discussed. However, each paper included in the review reflected the following claims about the mental function of physical activity:

#### 3.2.1 Self-esteem

Self-esteem is regarded as an important indicator of well-being; it connects with our health behaviors in daily life. The positive link between physical activity and self-esteem has been established. Several meta-analytic reviews have been conducted in this area, such as Wang et al. (2016), Molina-García et al. (2011), and Zheng et al. (2014), shown that physical activity has a positive influence on self-esteem regardless of physical activity type.

Wang et al. (2016) investigated the impact of a 4-week competitive taekwondo (CT) training in improving psychological well-being. Ten college students were randomly assigned to a training program which involved a 90-min CT exercise class twice per week for four weeks. Their symptoms were subsequently compared with those of a non-exercising control group, and it was concluded that this form of activity ameliorated symptoms.

While most researchers focus on differences between exercisers and non-exercisers, few studies reported result for man and women together. Another review examined leisure-time physical activity as a mental health promoting tool with conflicting results across samples of man and woman. Molina-García et al. (2011) indicated that male college students are more active and rated self-esteem higher than female college students. However, such findings may be influenced by cultural biases. The Spanish sample failed to account for how traditional gender role attitudes influence physical activity engagement and benefit perception. The phenomenon potentially linked to masculine athletic expression preferences within machismo culture.

#### 3.2.2 Depression

The diagnoses of depression have grown increasingly common among college students in recent years. A growing area of research focuses on the impact of physical activity on depression reduction (Chuan and Xiong, 2023; Yazici et al., 2016; Zhou and Guo, 2023). One empirical research confirms the correlation between physical activity and depression; the analysis of the obtained results indicates a negative correlation between these two variables. However, these are very weak correlations of r < 0.2 and can, therefore, only be taken as indicators of a positive or a negative correlation. Additionally, the weak correlation could suggest that while physical activity has a positive impact on mental health, it may not be the only or primary factor influencing depression levels.

Yong Zhou’s study also found that physical activity is negatively correlated with depression (Zhou and Guo, 2023). Different ways of linking physical activity with depression have been shown; however, using a chain mediation model to investigate this correlation in college students prevented us from exploring the corresponding physiological indicators. Limited evidence from Yazici’s study suggests that a tennis training program that involved a 90-min class once per week for 13 weeks has a beneficial effect on psychological well-being among young university students (Yazici et al., 2016).

Overall, the evidence for an association between PA and depression reduction based on controlled studies was inconsistent; the mechanisms of the effect of physical activity on depression require further study.

#### 3.2.3 Subjective well-being, happiness, and mood

A growing number of controlled studies are used to support the general benefits of physical activity on well-being. In a wide-ranging literature review, Blažević et al. (2021) indicate the correlation between physical activity and several indices of subjective well-being. He identifies the positive correlation between physical activity and good mood, positive well-being, and the negative correlation between PA and anxiety and depression. Hou (2022) confirms that physical activity has a positive impact on subjective well-being (assessed by life satisfaction, positive emotion, and negative emotion). It shows a greater difficulty in establishing a precise association between these two variables, the instruments used to measure well-being varied across all studies, limiting the ability to compare results.

Hou (2022) also compares the differences in subjective well-being between college students majoring in physical education and college students without majors. On the one hand, PE majors’ systematic exposure to structured, high-intensity training fundamentally differs from non-majors’ incidental activity patterns. This divergence in activity quality and context creates ecological validity challenges when comparing generic “physical activity” effects; On the other hand, the number of studies and sample sizes were insufficient to adequately analyze effects of physical activity. The small sample sizes reduce the precision of the estimates, making it difficult to generalize the results to a broader population.

Another finding provided empirical support for previous studies reporting the paramount role of PA in promoting subjective well-being. Furthermore, the differential effects of the different sports motivations are discussed. The results indicate that intrinsic motivations for sports are associated with higher SWB levels compared to extrinsic motivations (Jetzke and Mutz, 2020). Physical activities, which are closely connected to positive feelings and experiences like joy, flow, mastery, rush, team spirit, etc., can increase SWB positively. It is an interesting point to connect meanings, goals, and motivations related to sport with SWB indicators (satisfaction with life).

## 4 Conclusion

Due to a lack of experimental studies, few researchers have focused on the impact of PA on well-being of college students. However, college students also demonstrated a positive relationship between physical activity and well-being. It would be of utmost importance to promote physical activity in the university context in which lifestyles are being consolidated.

The majority of evidence in our study confirms that the impact of PA on well-being is positive. For example, in relation to depression, the results were in line with the ones obtained in a cross-sectional study of 5,451 men and 1,277 women by Hong et al. (2024), who showed a negative relationship between physical fitness level and depression. Although there is a lot of research on the topic of physical activity and well-being, we should realize that “physical activity” as a category includes a large variety of different types and settings of physical activities, which may vary in their contribution to a person’s well-being. Therefore, future investigations should prioritize mechanistic studies examining how distinct PA characteristics (frequency, social components, physiological demands) interact with well-being outcomes.
